# Supplementary material for: Hybrid Models and Biological Model Reduction with PyDSTool
Source: PLoS Comput Biol. 2012 Aug 9;8(8):e1002628. doi: 10.1371/journal.pcbi.1002628 (PMC3415397; doi:10.1371/journal.pcbi.1002628)
Supplement: Text S4 — Complete source code for the PyDSTool package (version 0.88.120504). Includes API documentation and help files linking to web pages. This file is identical to the current public release on Sourceforge.net. (ZIP) [file pcbi.1002628.s004.zip › PyDSTool/html/toc-PyDSTool.Toolbox.neuro_data-module.html]

xml version="1.0" encoding="ascii"?


neuro\_data


# Module neuro\_data

---

## Classes

burst\_feature  
 estimate\_spiking  
 geom\_feature  
 get\_burst\_active\_phase  
 get\_burst\_dc\_offset  
 get\_burst\_downsweep  
 get\_burst\_duration  
 get\_burst\_isi\_env  
 get\_burst\_num\_spikes  
 get\_burst\_passive\_extent  
 get\_burst\_peak\_env  
 get\_burst\_period\_info  
 get\_burst\_spikes  
 get\_burst\_trough\_env  
 get\_burst\_upsweep  
 get\_spike\_data  
 get\_spike\_model  
 spike\_envelope  
 spike\_feature  
 spike\_metric  

## Functions

find\_internal\_extrema  

---

[hide private]
